# Halogen-bonded shape memory polymers

**DOI:** 10.1038/s41467-022-34962-7

**Published:** 2022-12-05

**Authors:** Hongshuang Guo, Rakesh Puttreddy, Turkka Salminen, Alons Lends, Kristaps Jaudzems, Hao Zeng, Arri Priimagi

**Affiliations:** 1 Smart Photonic Materials, Faculty of Engineering and Natural Sciences, Tampere University, Korkeakoulunkatu 3, FI-33720 Tampere, Finland; 2 Tampere Microscopy Center, Tampere University, Korkeakoulunkatu 3, FI-33720 Tampere, Finland; 3 Department of Physical Organic Chemistry, Latvian Institute of Organic Synthesis, Riga, LV-1006 Latvia

**Keywords:** Polymers, Halogen bonding, Polymers

## Abstract

Halogen bonding (XB), a non-covalent interaction between an electron-deficient halogen atom and a Lewis base, is widely adopted in organic synthesis and supramolecular crystal engineering. However, the roadmap towards materials applications is hindered by the challenges in harnessing this relatively weak intermolecular interaction to devise human-commanded stimuli-responsive soft materials. Here, we report a liquid crystalline network comprising permanent covalent crosslinks and dynamic halogen bond crosslinks, which possess reversible thermo-responsive shape memory behaviour. Our findings suggest that I···N halogen bond, a paradigmatic motif in crystal engineering studies, enables temporary shape fixation at room temperature and subsequent shape recovery in response to human body temperature. We demonstrate versatile shape programming of the halogen-bonded polymer networks through human-hand operation and propose a micro-robotic injection model for complex 1D to 3D shape morphing in aqueous media at 37 °C. Through systematic structure-property-performance studies, we show the necessity of the I···N crosslinks in driving the shape memory effect. The halogen-bonded shape memory polymers expand the toolbox for the preparation of smart supramolecular constructs with tailored mechanical properties and thermoresponsive behaviour, for the needs of, e.g., future medical devices.

## Abstract (web summary)

Halogen bonding is widely adopted in organic synthesis and supramolecular crystal engineering but application of halogen bonding in the design of stimuli-responsive materials is challenging. Here, the authors report a liquid crystalline network that contains dynamic halogen-bond crosslinks and possesses reversible thermo-responsive shape memory behaviour.

## Introduction

Halogen bonding (XB) is a non-covalent interaction that has attained significant interest in the field of supramolecular chemistry during the past decades^1–3^. While XB is in many ways similar to hydrogen bonding (HB)^4–7^, the growing research interest is based on the differences rather than the similarities compared to HB. Four main differences between XB and HB can be identified^8^. First, the strength of a typical R − X···B halogen bond, where B is a neutral or negatively charged Lewis base and X is the electron-accepting donor attached to an electron-withdrawing moiety R, can be tuned by halogen substitution, decreasing in the order I > Br>Cl>>F^9^, and typically being weaker than corresponding HB. Second, the narrowly confined electropositive region along the R–X axis, known as the sigma-hole^10^, gives high directionality for XB, which has been utilized in supramolecular crystal engineering^11^ and the design of halogen-bonded liquid crystals^12,13^. Third, the large size of the halogen atom taking part in the supramolecular interaction (frequently iodine or bromine) may act as a heavy-atom perturber in the context of light-emissive materials, thereby promoting phosphorescence emission in organic materials^14,15^. Finally, unlike HB, XB is hydrophobic by nature, enabling applications such as anion sensing and transport in an aqueous environment^16–18^.

Despite the unique properties inherent to XB, in polymer sciences, it is still not widely utilized^19,20^. In the past years, encouraging examples have been reported wherein XB is used to control polymer self-assembly processes in both solid-state^21^ and solution^19,22^. When incorporated as chemical crosslinks, XB donor moieties can drive the formation of supramolecular gels^23,24^. A recent prime example describes dynamic XB-crosslinked polymer systems with the ability to self-heal^25,26^. XB has also been studied in light-responsive materials, where it promotes photocontrol over molecular alignment^27^ as well as light-driven mass migration and the formation of surface-relief gratings onto thin-film surfaces^28^. These earlier studies provoked us to ask the following questions: Instead of materials’ movements on surfaces, could dynamic XB crosslinks invoke also macroscopic shape changes of free-standing polymer films similar to hydrogen-bonded films in literature?^29,30^ If yes, can the unique attributes inherent to XB lead to novel functionalities that cannot be obtained using conventional shape-changing polymeric systems? To address these two questions, we turn our attention to shape memory polymers.

Shape memory polymers (SMPs) can change their configuration and macroscopic shape between permanent and temporary states^31,32^. The temporary shape is usually obtained through mechanical deformation under certain conditions, such as heating above the glass transition temperature (*T*_*g*_), followed by shape fixation upon cooling. The polymer maintains its temporary shape until a suitable external stimulus (*e*.*g*., temperature, chemicals, light) is applied to recover the permanent shape. The use of SMP products has grown and the market is currently experiencing a strong upward trend in, e.g., dentistry and household electronics, competing with those derived from shape memory alloys. Polymer-based smart textiles^33^, earplugs for noise cancellation, and nose pads for eye-glasses^34^ are some real-life applications of temperature-responsive SMPs available in the market.

Dynamic covalent bonds offer good thermal and chemical stability to SMPs. However, they often exhibit sluggish responsiveness to external stimuli^35^, necessitate the use of toxic catalysts preventing feasible biomedical applications^36^, and produce side reactions such as allyl–sulfide exchange^37^, Diels-Alder^38,39^, and redox reactions^40^. Thus, shape memory functions driven by non-covalent crosslinks, i.e., supramolecular SMPs, are becoming increasingly topical. Supramolecular SMPs involving dynamic HBs^41^, coordination bonds^42,43^, host-guest interactions^44,45^, and ionic bonds^46,47^ as the key driving force for the shape memory effect, have expanded the smart products space as well as fabrication methods with additional benefits such as rapid self-healing and reprocessability^48–50^.

Among a variety of stimuli-responsive supramolecular SMPs, their activation by human body temperature (～37 °C)^51,52^ for applications in drug delivery^53^, biomedical stents^54^, and implantable devices^31,55^ represents an emerging category of smart materials. Thermo-responsive supramolecular SMPs that operate at body temperature must combine two criteria: (i) the *T*_*g*_ lies in a range between room temperature (*ca*. 20 °C) and the body temperature and (ii) the materials sustain their mechanical strength under the operating conditions. Both criteria depend strongly on the nature of the non-covalent bonds used and the physical interactions between the molecular components, thereby making it challenging to achieve body temperature activated shape memory effect^56,57^. Nevertheless, strategies including the preparation of dual-crosslinking supramolecular SMPs^58,59^ and co-polymerization of co-monomers with SMP substrates^54^ to adjust molecular cohesive forces as well as thermal properties close to body temperature have been reported. However, these materials have disadvantages, such as the addition of plasticizers and low mechanical strength. In this context, the use of perfluorohalobenzene XB donors with the capability to form dynamic XB crosslinks as well as to impart hydrophobic interactions into a polymer network altering the *T*_*g*_, is deemed as an unexplored opportunity, which may preclude the need for additional co-monomers.

**Fig. 1:**
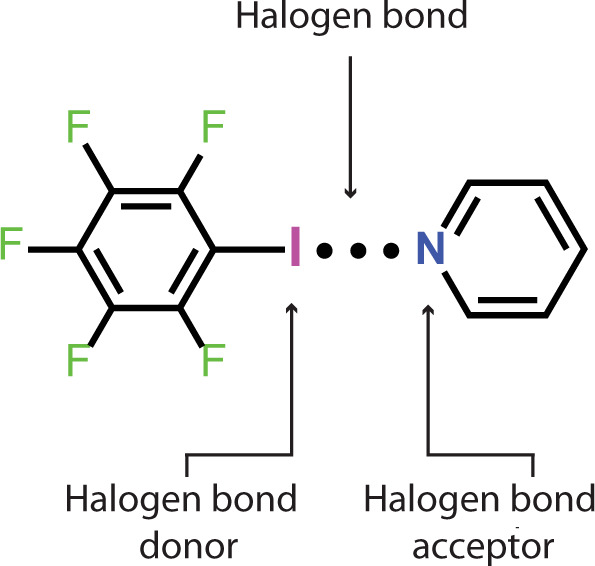
Halogen bond. General representation of a halogen-bonded complex depicting an I···N interaction.

**Fig. 2:**
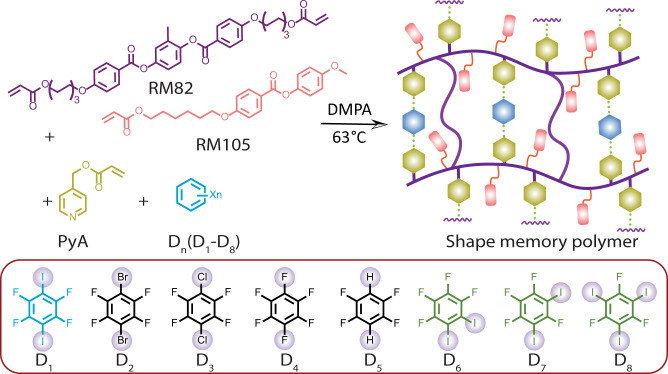
Chemical structures of shape memory polymers based on halogen bonds. Top: Synthesis route and proposed halogen-bonded shape memory polymeric structure of **PD**_**1**_. Bottom: The halogen/hydrogen bond donors (**D**_**1**_–**D**_**8**_). DMPA is a photoinitiator, 2,2-dimethoxy-2-phenylacetophenone.

Here, we show that XB between iodine on the aromatic ring and the nitrogen of a pyridine, as illustrated in Fig. 1, is responsible for the shape memory effect in a liquid crystalline network (LCN) depicted in Fig. 2. Fluorine substituents increase the sigma-hole strength of the iodine, hence strengthening the I···N XB. We have carried out an extensive and systematic experimental study on a series of polymer networks to shed light on the thermo-responsive shape memory performance driven by the XB. Furthermore, we demonstrate several material programming opportunities driven by heat stimulus close to human body temperature.

## Results

### Characterization of the halogen-bonded polymer networks

The materials used in this work are shown in Fig. 2. **P** is formed by photopolymerizing the mixture of RM82, RM105, and **PyA** in a 1:5:50 ratio, resulting in a loosely crosslinked liquid crystalline polymer network where the covalently grafted pyridine units act as XB acceptors. The LCN can be aligned upon stretching, as indicated by the birefringence observed from the cross-polarized imaging in Supplementary Fig. 1. **PD**_**1**_–**PD**_**8**_ are obtained by incorporating **D**_**1**_–**D**_**8**_ into the primary polymer network (2:1 ratio between **PyA**:**D**_**1–7**_ and 3:1 ratio between **PyA**:**D**_**8**_) (Supplementary Table 1). As the sigma-hole size decreases in the order I > Br>Cl>>F, only the heavier iodine and bromine are expected to form the I/Br···N interactions^10^. **PD**_**1**_–**PD**_**3**_, having a 180° angle between the two donor sites, allow us to compare the influence of halogen atom substitution (I vs. Br vs. Cl) on the mechanical and shape memory properties. **PD**_**4**_ and **PD**_**5**_ serve as secondary and tertiary references due to the absence of XB interactions. In **PD**_**6**_ and **PD**_**7**_, the angle between the XB donor sites is changed to 60° and 120°, respectively, while **PD**_**8**_ constitutes three donor sites at 120° angles. Across the samples **PD**_**1**_, **PD**_**6**_, **PD**_**7**_, and **PD**_**8**_, the angle between the iodine varies while the I···N interaction strength remains roughly the same.

**Fig. 3:**
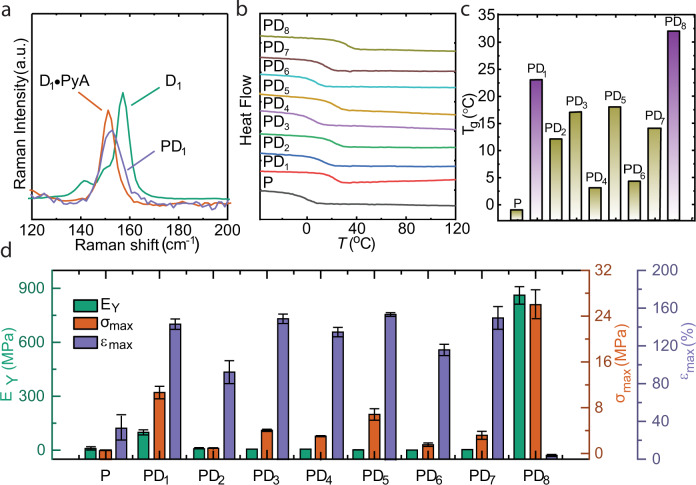
Characterization of the halogen-bonded polymer. **a** An overlay Raman spectrum of **D**_**1**_, **D**_**1**_**･PyA**, and **PD**_**1**_, indicating that halogen bonding results in C–I stretching band red shift. **b** DSC curves of **P** and **PD**_**1**_**-PD**_**8**_, **c** the *T*_*g*_ of **P** and **PD**_**1**_**-PD**_**8**_ obtained from (**b**). **d** Young’s modulus (E_Y_), fracture strain (ε_max_), and tensile strength (σ_max_) of **P** and **PD**_**1**_–**PD**_**8**_, Error bars represent the standard deviation obtained from three experiments.

We used Raman spectroscopy to study the C–X vibrational modes of the halogen-bonded polymer complexes^60–62^. Typically, the signal characteristic of uncomplexed C–X bond locates at 100–400 cm^−1^ and red shifts upon XB formation^63,64^. As shown in Fig. 3a and Supplementary Fig. 2, **D**_**1**_**･PyA** (a non-polymerized 1:2 mixture of **D**_**1**_:**PyA**) and **PD**_**1**_ demonstrate clearly the red shifts compared to plain **D**_**1**_, implying a weaker C–I bond force constant and n → σ* nature of the I···N interaction^65^. The red shift in **D**_**1**_**･PyA** is larger than in **PD**_**1**_, implying that other physical interactions occurring in the polymer network that are not present in **D**_**1**_**･PyA** may weaken the XB interaction. This trend, a larger red shift for a corresponding small-molecule-based XB complex than for a polymeric XB complex, is consistent with the previously reported C–I Raman frequencies of perfluoroiodobenzenes when complexed to small-molecule N-heterocycles^66^ and corresponding polymers^24^. In **D**_**2–5**_**･PyA** and **PD**_**2–5**_, the C–X/H frequencies are similar to the corresponding donors (Table 1 and Supplementary Figs. 3–6), indicating the absence of XB. In fact, for **D**_**4,5**_**･PyA**, the Raman signal was not observed at all due to the liquid-liquid phase separation of **D**_**4**_/**D**_**5**_ and **PyA** (Supplementary Figs. 7–9). This observation suggests that the I···N XB strengthens the supramolecular network and generates a crystalline material while the liquid-liquid phase segregation (Supplementary Figs. 8,9) only takes place when much weaker non-XB interactions, such as F···F, C–H···*π*, *π*-*π*_F_ and *π*_F_-*π*_F_, are present. **D**_**6**_ exhibits two distinct peaks at 223 and 233 cm^−1^. We speculate that this doublet Raman signal may arise from asymmetric stretching of C–I bonds and potentially type II XBs (Supplementary Fig. 14a)^67^. On the contrary, despite **D**_**7**_ and **D**_**8**_ have previously been shown to exhibit type II XBs in the solid state^68–70^, their corresponding C–I vibrations exhibit a singlet at 199 and 173 cm^-1^, respectively. Nevertheless, analogous to **D**_**1**_**･PyA** and **PD**_**1**_, the C–I frequencies of **D**_**6-8**_**･PyA** and **PD**_**6,8**_ are red shifted, implying longer C–I bond equilibrium distances and the assertion of I···N XB (Supplementary Figs. 10–12). Additionally, solid-state NMR was used to study the XB complexation in **D**_**1**_**･PyA** and **PD**_**1**_ and further information can be found in Supplementary Note 2.

**Table 1:** Experimental Raman frequencies^a^ for D_n_, D_n_ PyA, and PD_n_

Donor, D_n_	*v* (cm^−1^)	D_n_ PyA	*v* (cm^−1^)	PD_n_	*v* (cm^−1^)
D_1_	157	D_1_･PyA	151	PD_1_	153
D_2_	209	D_2_･PyA	210	PD_2_	211
D_3_	351	D_3_･PyA	352	PD_3_	352
D_4_	558	D_4_･PyA	–^b^	PD_4_	561
D_5_	747	D_5_･PyA	–^b^	PD_5_	747
D_6_	223/233	D_6_･PyA	223/230	PD_6_	221/230
D_7_	199	D_7_･PyA	194	PD_7_	–^b^
D_8_	173	D_8_･PyA	168	PD_8_	169

^a^The frequency error is no greater than 1−2 cm^−1^ for both strong and weak signals.

^b^No Raman signal observed.

One of the most effective ways to prepare body temperature-operating SMPs is to introduce fluorine groups into the polymer backbone to reduce the chain rotational flexibility through interchain F···F interactions^71^. Notable examples of such SMPs include polyvinylidene fluoride-based polymers^72^ and co-polymer blends^73^, and polyurethane urea^74^, all having *T*_*g*_ values close to body temperature and exhibiting good mechanical properties compared to their less substituted analogues. Inspired by the fluorine-based SMPs, we consider our **PD**_**n**_ systems as “semi-fluorinated” polymer networks and hypothesize that the inherent ability of the **D**_**n**_ molecules to manifest F···F contacts as well as the *π*_F_-core’s nature to manifest *π*-*π*_F_ and *π*_F_-*π*_F_ interactions^75^ would affect the *T*_*g*_ values and the mechanical properties of the polymer networks. We therefore determined *T*_*g*_ values and mechanical properties of all the polymers, despite the confirmation of I···N XBs only in **PD**_**1,6-8**_ by Raman analysis.

### Thermal and mechanical properties

The *T*_*g*_ of **P** and **PD**_**1-8**_ were determined with a differential scanning calorimeter (DSC) from second heating (heating/cooling rate of 10 °C/min). The *T*_*g*_ values range from −1 to 32 °C (Fig. 3b, c and Table 2). Compared to **P** (−1 °C), which comprises chains with a high degree of rotational freedom around the *σ*-bonds, the presence of I···N XBs significantly reduces the chain flexibility and increases the molecular cohesion in **PD**_**1**_, increasing the *T*_*g*_ to 23 °C. The *T*_*g*_ values of the secondary (**PD**_**4**_) and tertiary (**PD**_**5**_**)** references are larger than the primary reference (**P**), suggesting that they constitute stronger *π*-*π*_F_/*π*_F_-*π*_F_ interactions and promote stronger packing. **PD**_**5**_ has a higher *T*_*g*_ than **PD**_**4**_, which we attribute to the presence of acidic C–H protons and the associated weak hydrogen bonds they may form. A similar explanation^76^ has previously been used to support the high *T*_*g*_ values of semi-fluorinated compared to non-perfluorinated polymers^71^. **PD**_**6**_, with a 60° angle between the XB donors, has a lower *T*_*g*_ compared to **PD**_**7**_ (120° angle). We attribute this to preferential XB formation with pyridyls within the same polymer chain, resulting in lower crosslink density, while in **PD**_7_, both intra- and interchain XBs are formed (See Supplementary Fig. 14b). **PD**_**8**_, which consists of three XB donor atoms, has a *T*_*g*_ of 32 °C, indicating high crosslink density as compared to **PD**_6_ and **PD**_7_ (See Supplementary Fig. 14c).

**Table 2:** Glass transition temperature and Mechanical properties of P and PD_n_

Code	E_Y_ (MPa)	σ_max_ (MPa)	ε_max_ (%)	T_g_ (°C)
P	1.4 ± 0.1	0.5 ± 0.2	32 ± 12.5	−1.0
PD_1_	93.6 ± 14.2	10.5 ± 1.1	142 ± 5.5	23.0
PD_2_	0.8 ± 0.1	0.9 ± 0.1	91 ± 12.1	12.0
PD_3_	1.3 ± 0.1	3.9 ± 0.2	149 ± 5	17.0
PD_4_	1.2 ± 0.1	2.9 ± 0.1	134 ± 5	3.0
PD_5_	2.2 ± 0.5	6.7 ± 1	154 ± 2	18.0
PD_6_	0.9 ± 0.1	1.5 ± 0.3	115 ± 5	4.0
PD_7_	1.1 ± 0.2	3 ± 0.7	149 ± 12	14.0
PD_8_	863 ± 45	26 ± 2.6	2.4 ± 0.8	32.0

In addition to the *T*_g_ values of **P**-**PD**_n_ comprising RM82:RM105:PyA in 1:5:50 molar ratio (Table 2), we studied the influence of RM105 content on the *T*_g_ by changing the 1:*n*:50 molar ratio (*n* = 4,5,6; Supplementary Fig. 13). To our surprise, the *T*_g_ of **P** shifts to higher temperatures for both *n* = 4 and *n* = 6 as compared to *n* = 5. This seems to indicate that, by varying the concentration of RM105, the chains of **P** assume new molecular alignments that restrict molecular motion and lead to an increase in *T*_g_. For **PD**_1_-**PD**_8_, the *T*_g_ was not significantly affected by the RM105 content, indicating that RM105 has no additional strengthening effect on the supramolecular polymer network. Finally, it is important to note that only **PD**_1_ and **PD**_8_ present *T*_g_ values that fit into the critical operation window between room temperature and body temperature (Fig. 3c).

We next studied the mechanical properties of the **PD**_**n**_. The tensile behaviour is determined by uniaxial stretching of **P** and **PD**_**n**_ thin films (16 × 2 × 0.1 mm^3^), and the results are given in Fig. 3d. The reference polymer **P** has a low elastic modulus of 1.4 MPa, tensile strength of 0.5 MPa and fracture strain of 32%. On the other hand, **PD**_**1**_ exhibits an elastic modulus of 93.6 MPa, tensile strength of 10.5 MPa, and fracture strain of 142%. This indicates an increased chain rigidity due to the I···N XB. Conversely, **PD**_**2-5**_ films are soft at room temperature and have low tensile strengths, while the fracture strains remain at a reasonably high level, between 91 and 149%. We attribute this to the absence of XBs, as supported by the Raman analysis. **PD**_**8**_ exhibits the highest elastic modulus (863 MPa) and tensile strength (26 MPa) of the systems studied. However, it also has the lowest fracture strain, only 2.4%, making it brittle. This result provides additional support to our hypothesized structures in Supplementary Fig. 14, implying that the number of I···N XB crosslinks indeed is lower in **PD**_**6/7**_ than in **PD**_**1/8**_.

We also examined the effect of XB donors on the mechanical properties of **PD**_n_ via changing the **PyA**:**D**_n_ ratio from 2:1 to 1:1 (and also to 3:2 for **PD**_**8**_), maintaining the RM82:RM105 ratio at 1:5. In general, the elastic modulus, tensile strength, and fracture strain of polymers with **PyA**:**D**_**n**_ comprising 2:1 ratio are larger compared to **PyA**:**D**_n_ with 1:1 molar ratio (Supplementary Fig. 15 and Table 2). We believe that this can be attributed to the combination of (i) reduced crosslinking density due to the excess of **D**_**n**_ (especially for the case of **D**_1_) and (ii) the presence of additional weak interactions such as halogen···halogen, C–X···π and *π*-*π*_F_ contacts, which destruct the stable halogen-bonded supramolecular network. For **PD**_1_, Young’s modulus and fracture strain decreased from 94 to 0.7 MPa and 142 to 109%, respectively, indicating the dramatic impact of excess *π*-deficient systems on the halogen-bonded supramolecular network. In the case of **PD**_**8**_, Young’s modulus decreased from 863 (3:1 **PyA**:**D**_**8**_) to 600 (3:2) to 440 (1:1) MPa, while the corresponding fracture strains were 2.4, 21, and 53%, respectively, suggesting complex non-covalent bonding mechanisms that regulate the macroscopic properties of **PD**_**8**_. It should be noted that –O– ether, carbonyl oxygen, as well as π-carbons may also act as weak electron donors. These respective groups may engender C–X···O–C^77,78^, C–X···O = C^6,79^, and C–X···C_Ar_^80^ interactions and contribute to the cohesion of the chemical system.

Based on the above results, we conclude that **P**, as well as all the two-component donor-acceptor polymers except **PD**_**1**_, are weak and possess similar tensile strength profiles—they are not suitable for shape memory purposes. The I···N XB increases the mechanical strength of the polymer networks. **PD**_**1**_, with 180° between the two XB donor sites, combines relatively high modulus and fracture strain, while **PD**_**8**_ is too brittle for application as SMPs. Therefore, **PD**_**1**_ is deemed the most suitable shape memory material and is used in further experiments.

### Shape memory properties

**Fig. 4:**
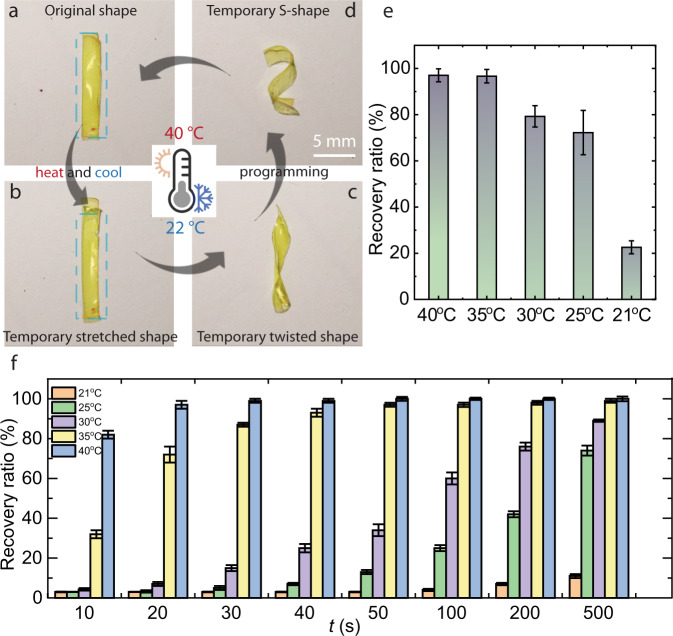
Thermo-responsive shape memory effect. **a**–**d** Photographs illustrating a shape recovery process of **PD**_**1**_ (sample dimensions: 20 × 1.0 × 0.1 mm). **e** Total shape recovery ratio at different temperatures after 1 h and **f** a plot depicting shape recovery ratio estimated at different times and temperatures. Error bars represent the standard deviation obtained from three experiments. The scale bar is 5 mm for all images.

A longitudinal stretching (20%) of a **PD**_**1**_ film (Fig. 4a) at elevated temperature (40 °C) and cooling to room temperature leads to a temporary deformed structure (Fig. 4b). Further processing at 40 °C and subsequent cooling provides the sample with other temporary shapes like the twisted and “S” shapes shown in Fig. 4c, d. The temporary deformations return to the original shape upon re-heating the sample. The shape memory behaviour presumably involves the following mechanism: at elevated temperatures, the halogen-bonded supramolecular network is mobile and at high entropy state, and the strip becomes deformable. During the cooling process, the XBs are re-established and allow shape fixation at the temporary configuration. This confronting process instils strain energy in the covalently crosslinked network at the temporary states. New heating of the strip retains mobility and provides freedom for molecular movements, releasing the stored strain energy for the structure to return to its original state. We evaluated the shape recovery ratio at different temperatures and recorded 52 s for 96% recovery at 35 °C and 24 s at 40 °C (Fig. 4e, f and Supplementary Fig. 16), indicating that at a higher temperature, larger proportions of the XB domains become mobile and are involved in the release of the stored energy, which leads to shape recovery in a shorter time span. The estimated shape fixation ratio of **PD**_**1**_ is 97% after 5 min to the temporary shape. The shape recovery time at 35 °C being <1 min, we next proceed to demonstrate a prototype SMP material operating close to body temperature, triggered by human palm and physiological media [Dulbecco’s phosphate-buffered saline (DPBS) with Earl’s salts and minimum essential medium (MEM)] at 37 °C.

**Fig. 5:**
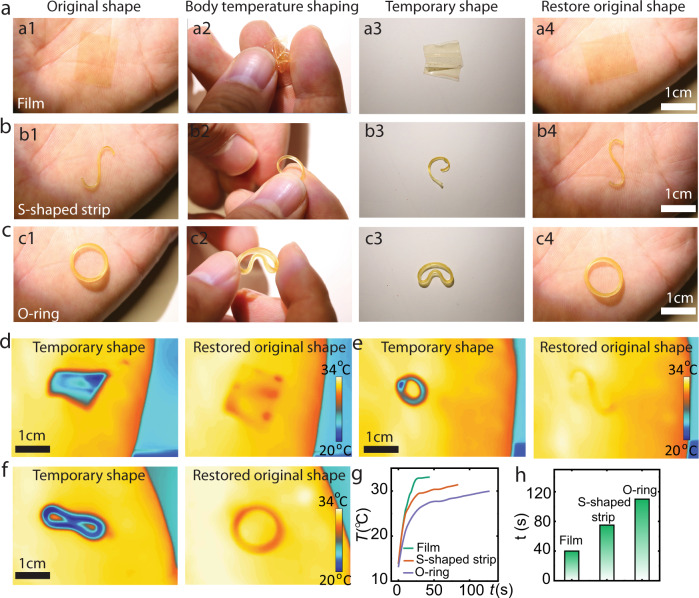
Body temperature triggered shape memory effect. Photographs of human palm-stimulus triggered shape memory processes of **a** film, **b** S-shape strip, and **c** the O-ring. a1–a4 represent the shape and recovery process of the film. b1–b4 represents the shape and recovery process of the S-shape strip and c1–c4 represents the shape and recovery process of the O-ring. Infrared thermal images of temporary and permanent shapes of **d** film, **e** S-shape strip, and **f** O-ring. **g** Temperature vs recovery time plot of the film (green line), S-shape strip (brown line), and O-ring (purple line). **h** Recovery time of different temporary shapes.

Figure 5 shows three examples of shape programming by human body temperature, based on the halogen-bonded **PD**_**1**_ network: a plain film (Supplementary Movies 1,2), an S-shaped strip (Supplementary Movies 3, 4), and an O-ring (Supplementary Movies 5, 6). The sample thicknesses are 100 μm (film), 1 mm (S-shaped strip), and 2 mm (O-ring), which affects the thermal capacity and the heating-cooling speed of the samples. In all cases, a bare palm (*ca*. 35 °C) suffices to attain shape memory programming: initial deformation by fingers, temporary shape fixation on top of a table at room temperature, and shape recovery by re-placing the structure on a hand palm. Infrared camera images (Fig. 5d–f) reveal the temperature evolution during the shape recovery process (Fig. 5g). Evidently, the heat released by the bare palm, although mild, is enough to recover the polymer structure after 40 s for a film, 78 s (S-shape), and 117 s for an O-ring (Fig. 5h). Samples configured to various other geometries are given in Supplementary Fig. 17.

Achieving the robust and high-fidelity shape memory effect of **PD**_**1**_ requires the halogen-bonded network to undergo multiple cycles of shape retention without deterioration in its performance. This was probed by carrying out 100 consecutive cycling experiments using the O-ring sample (Fig. 5c). As shown in Supplementary Fig. 18, no signs of fatigue are observed over the 100 cycles studied. Similar results were obtained also for the film geometry (Fig. 5a). We also confirmed that the stress-strain profile of **PD**_**1**_ remains unchanged after the 100 shape memory cycles (Supplementary Fig. 19a). These results highlight the ability of XB to introduce elasticity to the shape memory polymer during cycling, leading to robust cyclic deformation without deterioration in performance. Also, several weak van der Waals forces, such as C–H···I and C–I···*π*, which can be readily broken and reformed, may ensure molecular motion relevant to restoring the original shape during the memory process. We believe that the role and mechanism of these directionless non-covalent bonds in **PD**_1_ might be similar to the mechanism in elastic organic crystals of polyhalogenated N-benzylidineanilines reported by ref. 81.

We also carried out XB donor exchange experiments by soaking the **PD**_**1**_ film for 4 h in dichloromethane containing 1 M of **D**_2_. As shown in Supplementary Fig. 19b, the mechanical properties of **PD**_**1**_ were severely affected by this procedure, becoming similar to the mechanical properties of **P**. This suggests that **D**_**1**_ is released to dichloromethane instead of being replaced by **D**_2_. We believe, however, that an XB donor that is structurally and geometrically similar to **D**_1_ but capable of forming stronger I···N XB interactions could undergo the donor exchange.

**Fig. 6:**
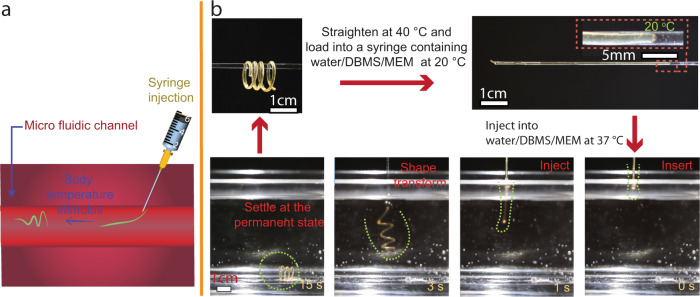
Application demo of halogen-bonded shape memory polymer. **a** A schematic representation of shape recovery of an injected temporary shape by body temperature stimulus. **b** Photographs illustrating the original coil shape (top left) and the syringe needle loaded with a straightened temporary shape, and the injection process of the temporary shape into 37 °C distilled water, which recovers the original coil shape. Sample dimensions: length 14 cm, 1 mm.

The major benefit brought out by the halogen-bonded SMP is that the material allows a facile manipulation step, using human skin temperature^34^, to transform a plain structure into a 3D shape. Or vice versa—a complex structure can be fabricated as the original state and later shape-programmed into a plain geometry. This concept can be attractive for applications in the biomedical field because of the possibility to be stored and delivered into a microfluidic device, or in the long term, into a patient, in a temporary shape and revert to a complex permanent shape in response to body fluids’ temperature, as demonstrated schematically in Fig. 6a.

We prepared a coil-shaped SMP filament through moulding-assisted polymerization. Upon shape programming at 40 °C, the coil was straightened to linear geometry and loaded into an injection needle (inner diameter 1.4 mm) filled with 20 °C water (Fig. 6b and Supplementary Movie 7). The coil structure is chosen as the prototype demonstration because this geometry can exhibit the propelling capacity under external field control^82–84^. Once the linear SMP filament is injected into 37 °C distilled water, the original coil shape is retained. Importantly, the aqueous environment does not pose any challenge to the shape memory effect, due to the hydrophobic nature of XB. Besides the shape memory effect in water, successful shape transformations were demonstrated in biological media, namely DPBS and MEM (Supplementary Figs. 20, 21).

## Discussion

A series of supramolecular shape memory polymers, crosslinked with both permanent covalent bonds and reversible supramolecular (halogen) bonds have been prepared in a one-pot synthesis, with the target of modulating only the latter using haloperfluoroaromatic, small-molecule halogen-bond donors. We have shown that I···N halogen-bond crosslinks are an essential element for the shape fixity and shape recovery of the observed shape memory behaviour. Weak halogen-bond donors such as 1,4-dibromotetrafluorobenzene and 1,4-dichlorotetrafluorobenzene, or sterically congested linkers such as 1,2-diiodotetrafluorobenzene, 1,3-diiodotetrafluorobenzene, or 1,3-triiodotrifluorobenzene produce either weak or brittle materials and therefore are not suitable for shape memory applications. The optimal bifunctional halogen-bond donor for a reversible shape memory effect is 1,4-diiodotetrafluorobenzene, and the corresponding polymer network exhibits excellent mechanical and thermal properties, rendering it operational at body temperature. We demonstrate the halogen-bond-driven shape memory effect upon exposure to the human hand, water, and physiological media at human body temperature. Our results demonstrate that halogen bond donors must be carefully chosen to achieve a delicate balance between crosslink density, mechanical performance, and deformation ability, opening new avenues for the design of halogen-bonded stimuli-responsive, shape-changing materials.

## Methods

### Materials

All reagents and chemicals were used as received without further purification. 1,4-Bis-[4-(6-acryloyloxyhexyloxy)benzoyloxy]-2-methylbenzene (99%, RM82) and 4-methoxybenzoic acid 4-(6-acryloyloxy-hexyloxy) phenyl ester (99%, RM105) were purchased from Synthon Chemicals GmbH & Co. The pyridyl acceptor (PyA) was synthesized according to the literature (See Supplementary Figs. 22, 23)^85^. 1,4-Diiodotetrafluorobenzene (**D**_**1**_) and 1,2-diiodotetrafluorobenzene (**D**_**6**_) were purchased from Fluorochem Ltd., 1,3,5-trifluoro-2,4,6-triiodobenzene (**D**_**8**_) and 1,3-diiodotetrafluorobenzene (**D**_**7**_) from Apollo Scientific, 1,4-dicholorotetrafluorobenzene (**D**_**3**_) from Synquest Laboratories, hexafluorobenzene (**D**_**4**_), 1,4-dibromotetrafluorobenzene (**D**_**2**_) and 1,2,4,5-tetrafluorobenzene (**D**_**5**_) from TCI Chemicals Europe, and the photoinitiator, 2,2-dimethoxy-2-phenylacetophenone (DMPA) from Sigma Aldrich.

### Samples preparation of PD_1_ for shape memory demonstrations

#### Film preparation

Two clean glass slides (2.5 × 3.0 × 0.2 cm) were spin-coated with 5% Poly(vinyl alcohol) solution and oven baked at 100 °C for 10 min. The glass slides were glued together using UV glue (Norland, NOA65) by placing them in between 100 μm spacer particles. The polymerizable mixture [0.02 mmol RM82, 0.1 mmol RM105, 1 mmol PyA, 0.5 mmol **D**_**n**_, and 0.02 mmol DMPA] were placed in a 20 mL glass vial and heated to 85 °C until all the components melted into a clear liquid. The molten mixture was introduced into the cell through capillary action, followed by slow cooling to 60–65 °C and subsequent irradiation under UV light (360 nm, 180 mW/cm^2^, 20 min). Strips of dimensions 20 × 2 × 0.1 mm were cut and peeled out using a surgical blade, and were used for shape memory demonstrations.

#### Preparation of S-shape and O-ring samples

The above-prepared molten photopolymerizable mixture was poured into a transparent S-shaped (outer diameter: 1.4 mm, inner diameter: 1 mm, and length: 50 mm) or cylindrical (outer diameter: 16 mm, inner diameter: 15 mm, and height: 2 mm) tube and allowed to solidify. The solidified parts were ejected out by cutting the tubes. To prepare the O-ring for shape memory demonstrations, the two ends of the tube were hand-pressed together to create a seal.

#### Preparation of coil for injectable robotics

The molten mixture [0.02 mmol RM82, 0.1 mmol RM105, 1 mmol PyA, 0.5 mmol **D**_**1**_, and 0.02 mmol DMPA] was poured into a transparent tube (length, 15 cm; outer diameter 1.4 mm) that was coiled around a 3 mm NMR tube, and subsequently photopolymerized at 60 °C (360 nm, 180 mW/cm^2^, 20 min irradiation from both sides) to yield the halogen-bonded **PD**_**1**_ polymer. The solidified coil was removed after cooling by cutting the tube.

#### Instrumentation and characterization details

Each specimen was placed on a palm and the shape transformations of the corresponding samples were recorded by using a Canon 5D Mark III camera equipped with a 100 mm lens (images and Supplementary Movies 1–7). Thermal images were recorded with an infrared camera (FLIR T420BX) equipped with a close-up (2×) lens. CoolLED pE-4000 was used as the UV light source (365 nm) for polymerization. DSC measurements were performed with a NETZSCH DSC 214 polyma instrument at a heating/cooling rate of 10 °C/min. The measurements were performed using a 7−12 mg sample under a 1 bar nitrogen atmosphere (flow rate of 20 mL/min) at the temperature range between −50 and 150 °C. The thermal properties were analyzed using the DSC data of the second, third, and fourth heating. Stress−strain curves were determined by using a homemade tensile tester using 100-μm-thick films (stretching speed of 0.05 mm/s). Raman spectra were obtained using a Renishaw Raman Microscope setup equipped with a 785 nm laser. The spectra were collected in the range of 110–1500 cm^−1^ (See Supplementary Figs. 2–12).

#### Shape recovery tests

Shape fixation [(*θ*_fixed_/*θ*_max_) × 100] and recovery ratio [(*θ*_max_*–θ*_*i*_)/*θ*_max_) × 100] were estimated by following literature procedures^86^. Samples of dimensions 20 × 1.0 × 0.1 mm were heated on a 40 °C hot plate and bent to an angle *θ*_max_ by applying an appropriate force. The bent shape was cooled to room temperature under the applied external force, after which the force was removed. The angle of the room temperature fixed shape is denoted as *θ*_fixed_. The fixed shape was placed on a hot plate at different temperatures and the bending angle (*θ*_*i*_) was recorded at different times, as illustrated in Supplementary Fig. 16. Kinovea program was used to monitor the motion of the temporary shape, yielding distance and time data at 1/30 s time intervals as coordinates for x- and y-axis. The angle is calculated by using tan*θ* = y/x.

## Supplementary information

Supplementary Information

Description of Additional Supplementary Files

Supplementary Movie 1

Supplementary Movie 2

Supplementary Movie 3

Supplementary Movie 4

Supplementary Movie 5

Supplementary Movie 6

Supplementary Movie 7

## Data Availability

The authors declare that the data that supports the findings of this manuscript can be found in the Supplementary Information and are available free of charge or available from the corresponding author upon request. Source data are provided with this paper.

## Source data

Source Data
