# Supplementary material for: Halogen-bonded shape memory polymers
Source: Nat Commun. 2022 Dec 5;13:7436. doi: 10.1038/s41467-022-34962-7 (PMC9723116; doi:10.1038/s41467-022-34962-7)
Supplement: Supplementary file 1 — Supplementary Information [file 41467_2022_34962_MOESM1_ESM.pdf]

Supplementary Information for

Halogen-Bonded Shape Memory Polymers

Hongshuang Guo,[1] Rakesh Puttreddy,[1*] Turkka Salminen,[2] Alons Lends,[3] Kristaps Jaudzems,[3] Hao Zeng,[1] and Arri Priimagi [1*]

Affiliation:

[1]Smart Photonic Materials, Faculty of Engineering and Natural Sciences, Tampere University, Korkeakoulunkatu 3, FI-33720 Tampere, Finland.

[2]Tampere Microscopy Center, Tampere University, Korkeakoulunkatu 3, FI-33720 Tampere, Finland.

[3]Department of Physical Organic Chemistry, Latvian Institute of Organic Synthesis, Riga, LV-1006, Latvia.

**Correspondence to: arri.priimagi@tuni.fi, rakesh.puttreddy@tuni.fi

This PDF file includes:

Supplementary Fig. S1 to S21
Materials and Methods

Additional supplementary material for this manuscript includes:

Supplementary Video 1-7

Supplementary Table 1. Molar ratio of substrates used during the preparation of **P** and **PD$_n$** films.

Sample	RM82	RM105	PyA	D$_n$	DMPA
**P**	1	5	50	0	1
**PD$_1$**	1	5	50	25	1
**PD$_2$**	1	5	50	25	1
**PD$_3$**	1	5	50	25	1
**PD$_4$**	1	5	50	25	1
**PD$_5$**	1	5	50	25	1
**PD$_6$**	1	5	50	25	1
**PD$_7$**	1	5	50	25	1
**PD$_8$**	1	5	50	17	1

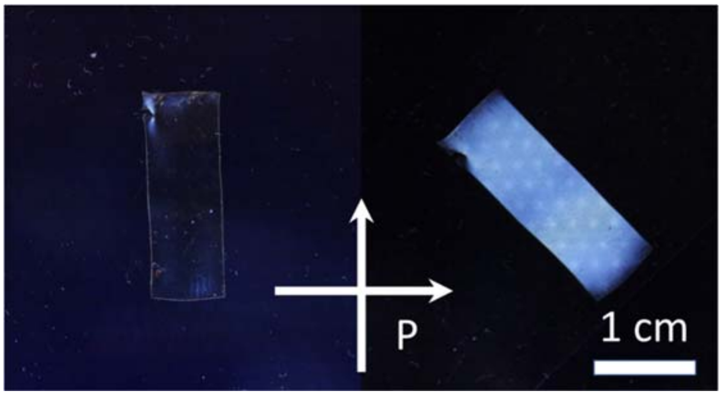

Supplementary Figure 1. Cross-polarized image of the halogen-bonded liquid crystal network upon 5% stretching. The arrows indicate the polarizer/analyzer directions.

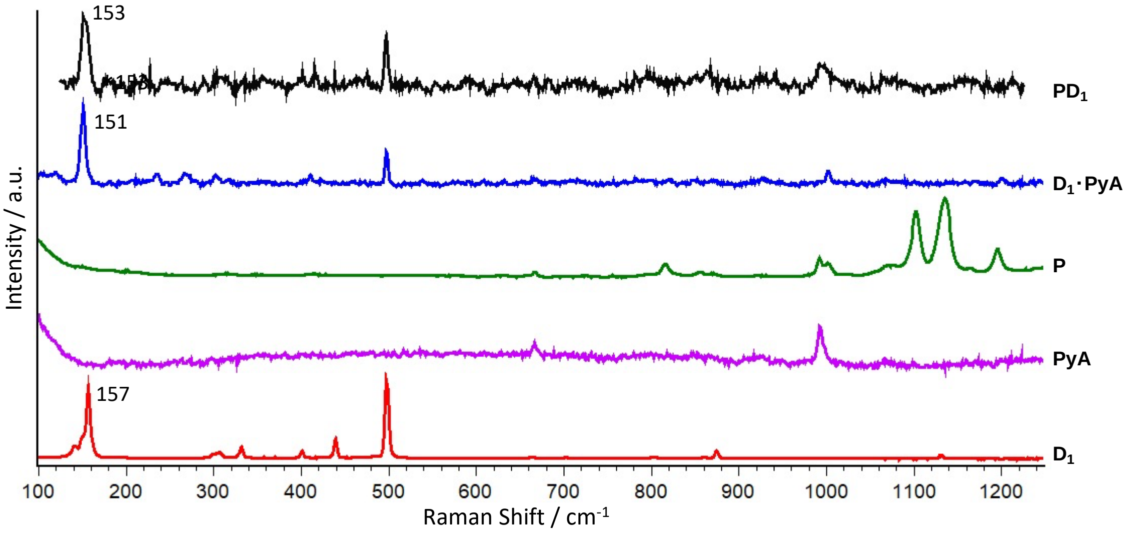

Supplementary Figure 2. Raman stack spectra of $\mathbf{D_1}$-$\mathbf{PD_1}$.

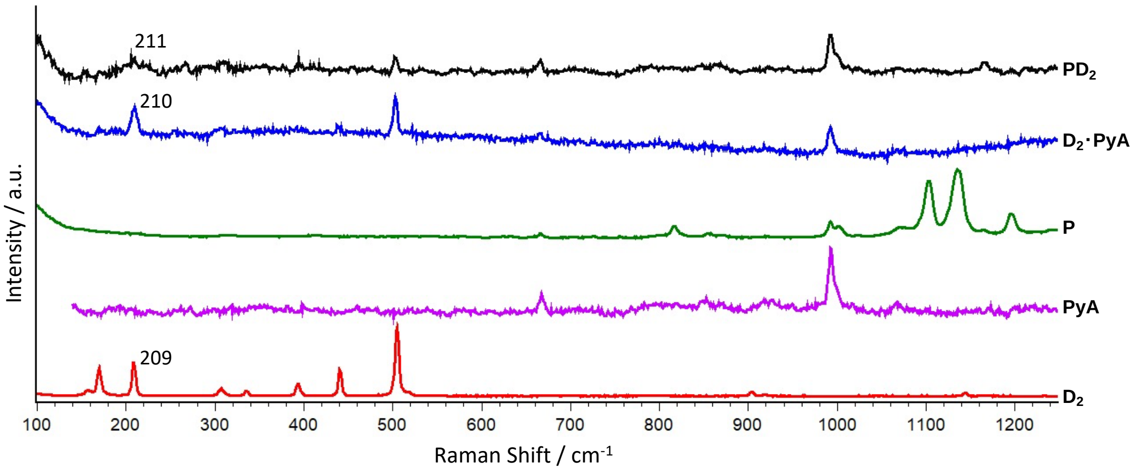

Supplementary Figure 3. Raman stack spectra of $\mathbf{D_2}$-$\mathbf{PD_2}$.

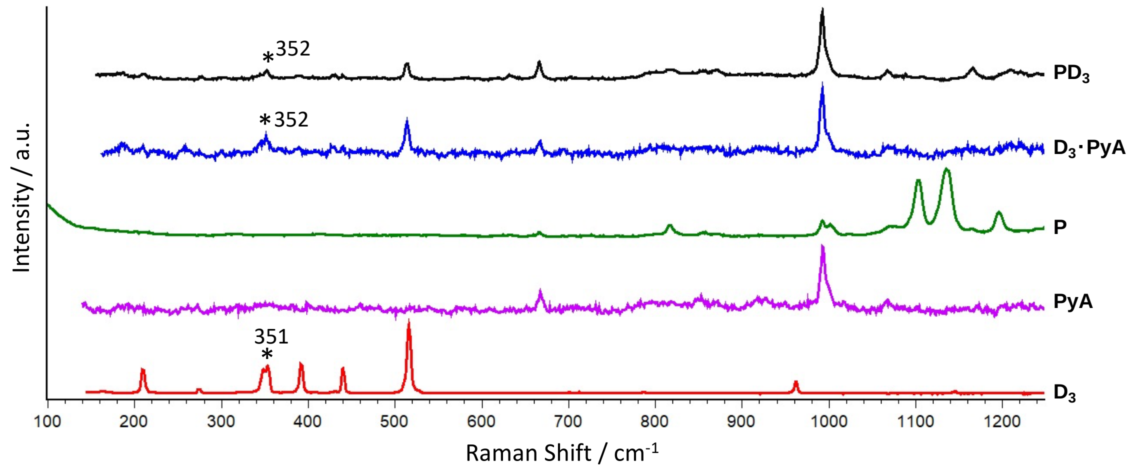

Supplementary Figure 4. Raman stack spectra of **D$_3$-PD$_3$**.

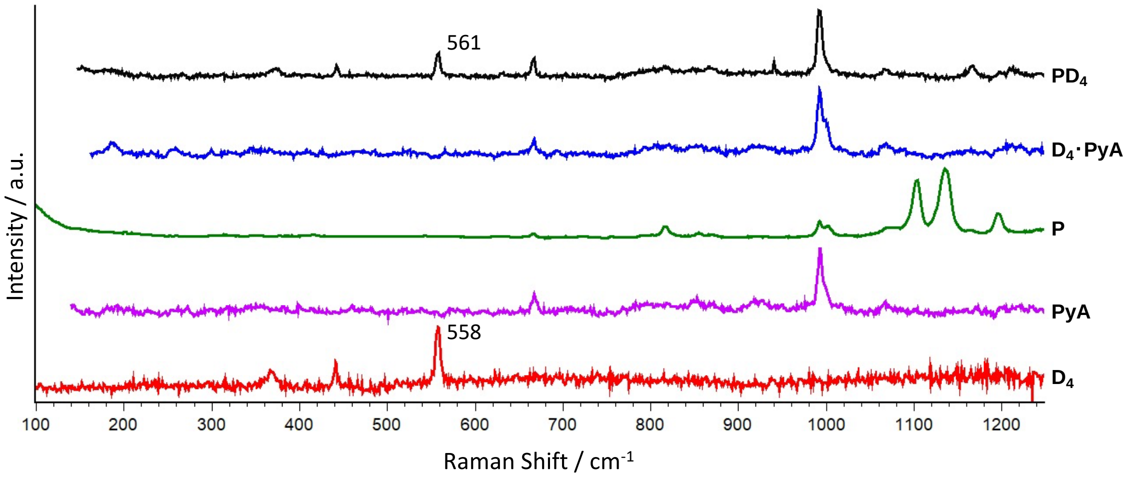

Supplementary Figure 5. Raman stack spectra of **D$_4$-PD$_4$**.

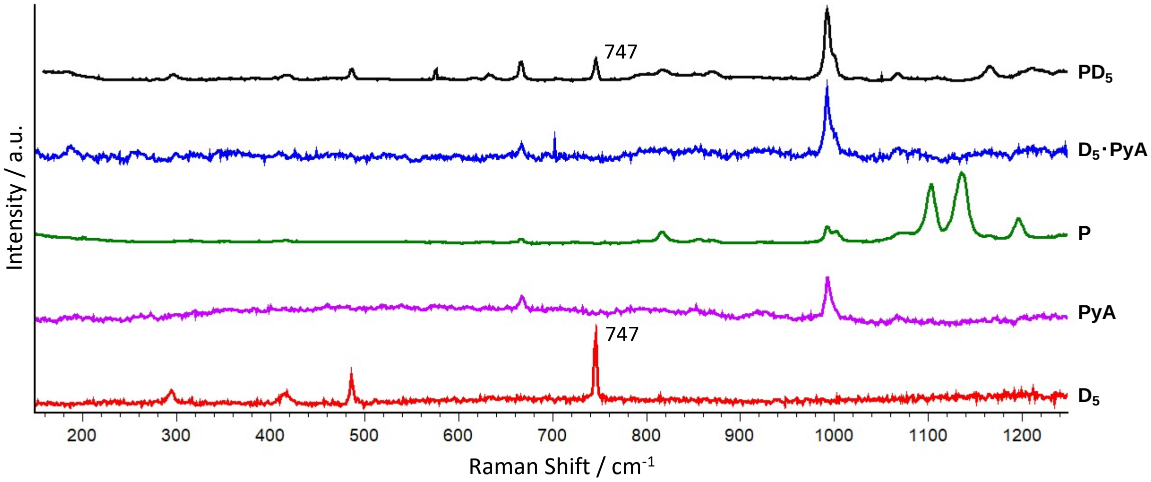

Supplementary Figure 6. Raman stack spectra of **D$_5$-PD$_5$**.

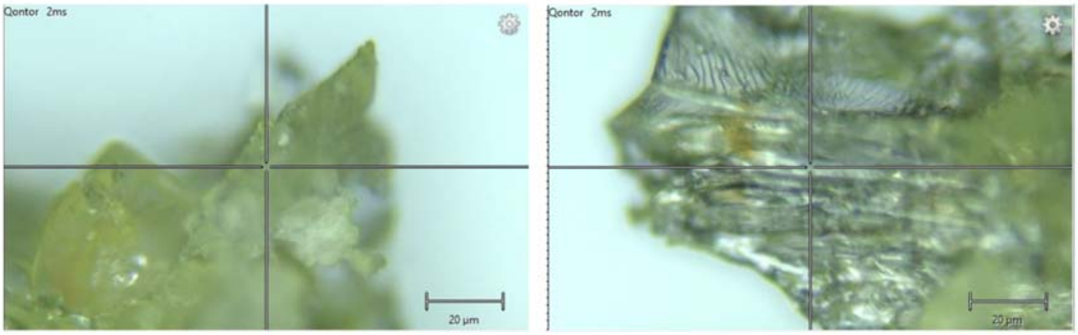

Supplementary Figure 7. Photographs of crystalline **D_1•PyA**. Note: The I···N halogen bonding between **D_1** and **PyA** produces strong supramolecular interactions. Hence, unlike **D_4•PyA** and **D_5•PyA** (Supplementary Figures 8 and 9), **D_1•PyA** is a crystalline sample.

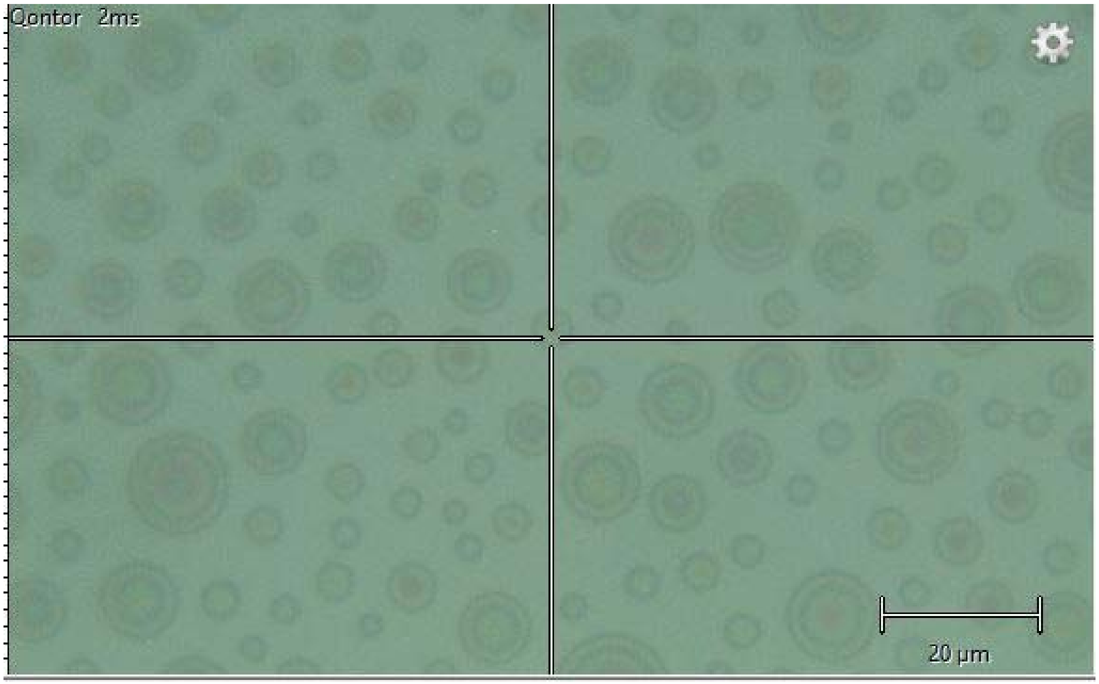

Supplementary Figure 8. Clustering of droplets of **D_4•PyA**.

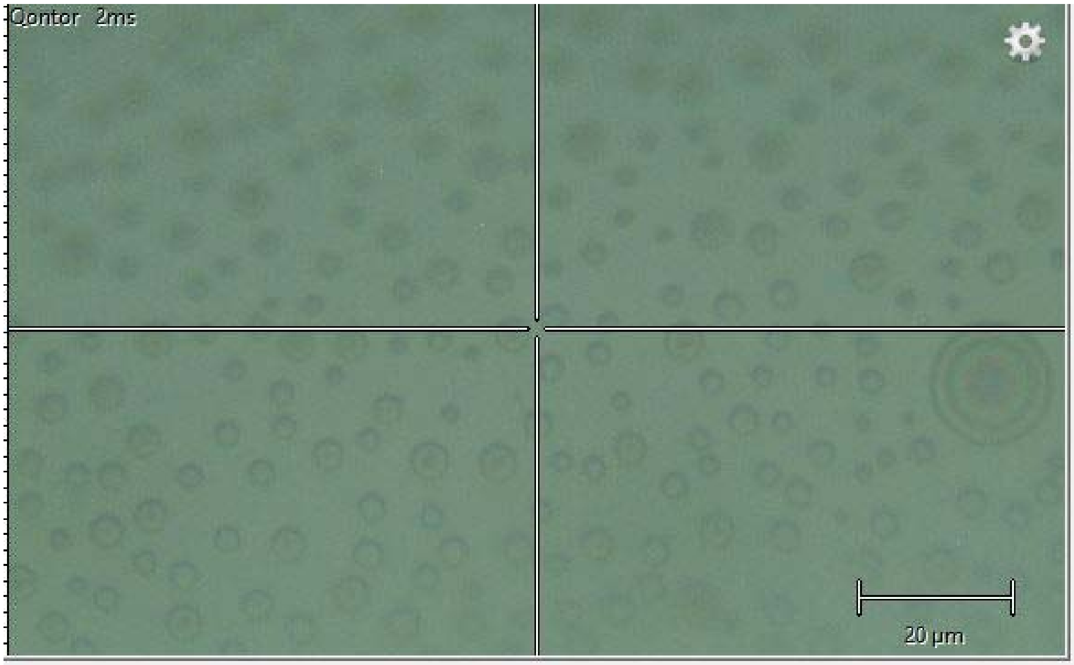

Supplementary Figure 9. Clustering of droplets of **D_5·PyA**.

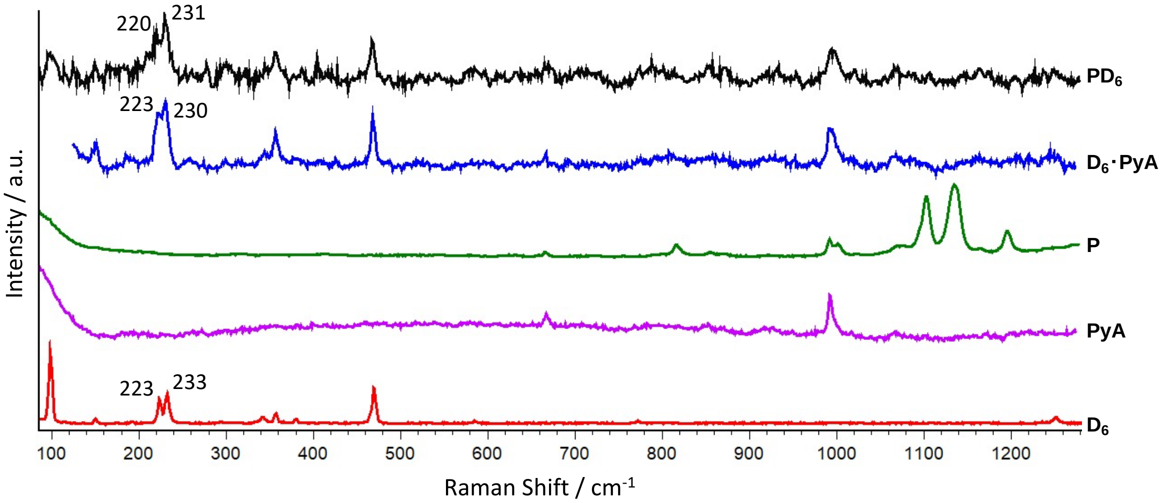

Supplementary Figure 10. Raman stack spectra of **D_6-PD_6**.

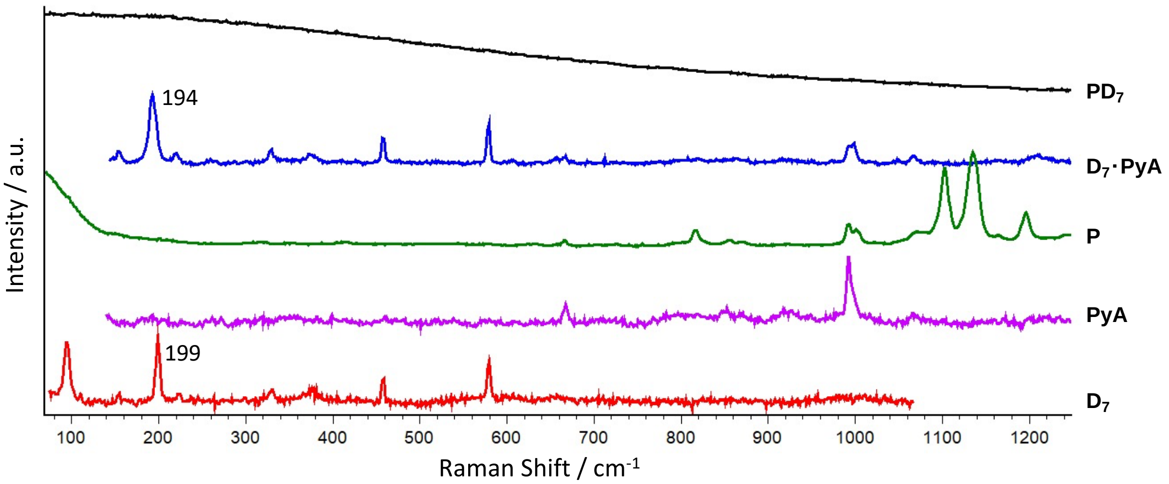

Supplementary Figure 11. Raman stack spectra of **D$_7$-PD$_7$**.

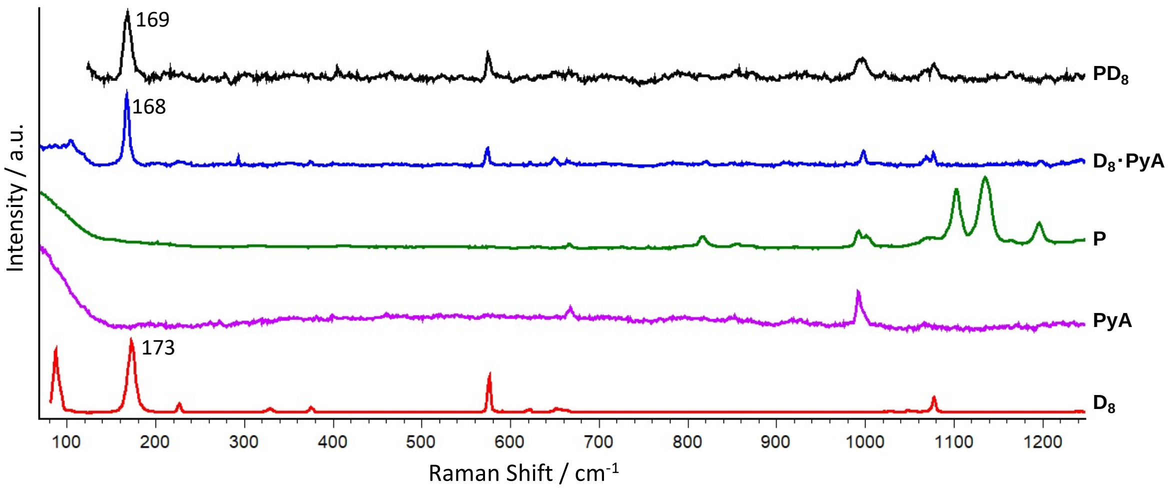

Supplementary Figure 12. Raman stack spectra of **D$_8$-PD$_8$**.

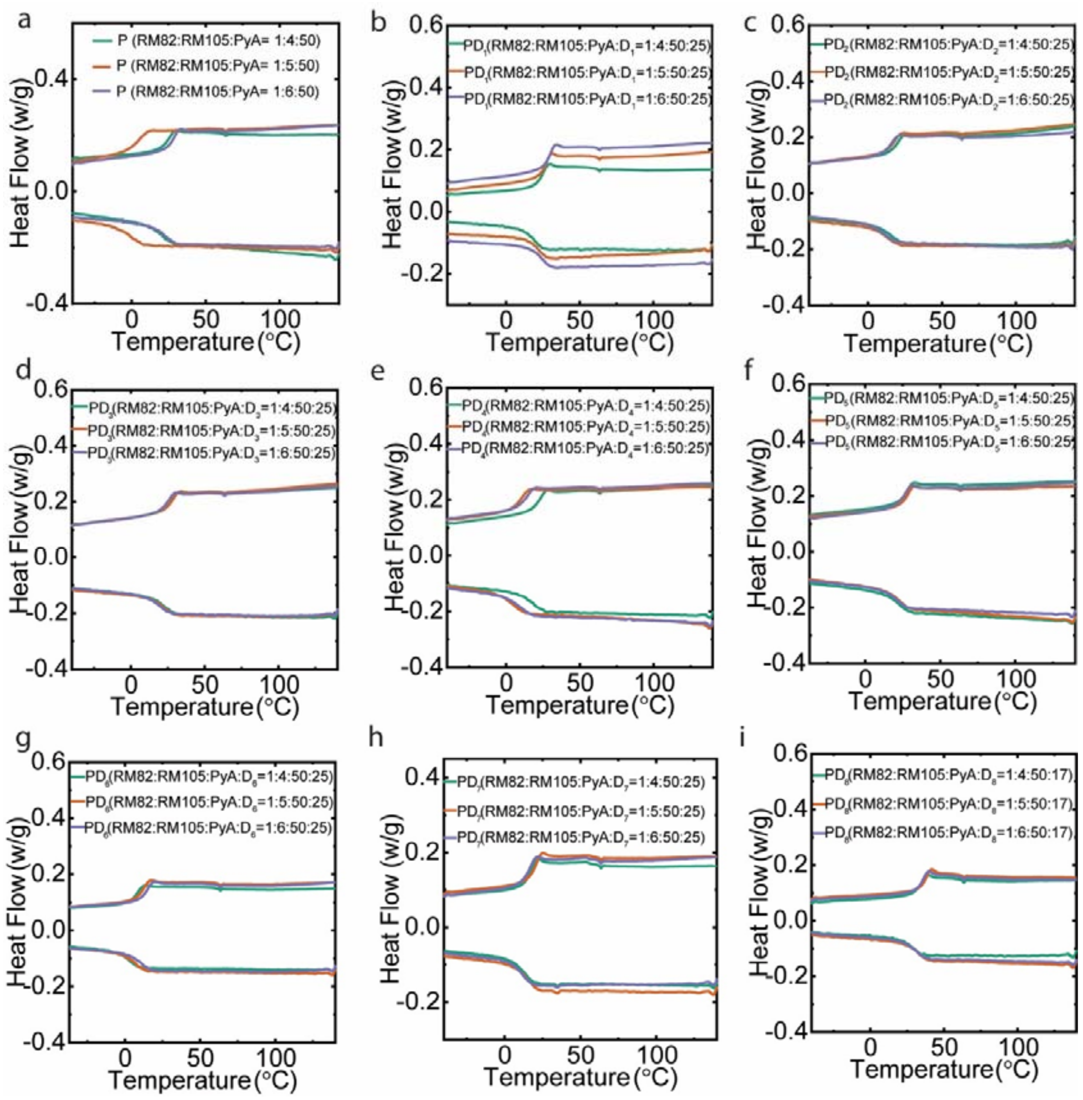

Supplementary Figure 13. (a)-(i) DSC curves of **P** and **PD$_1$-PD$_8$** with varying RM105 content. The thermal properties were analyzed using the DSC data of the second cooling and heating.

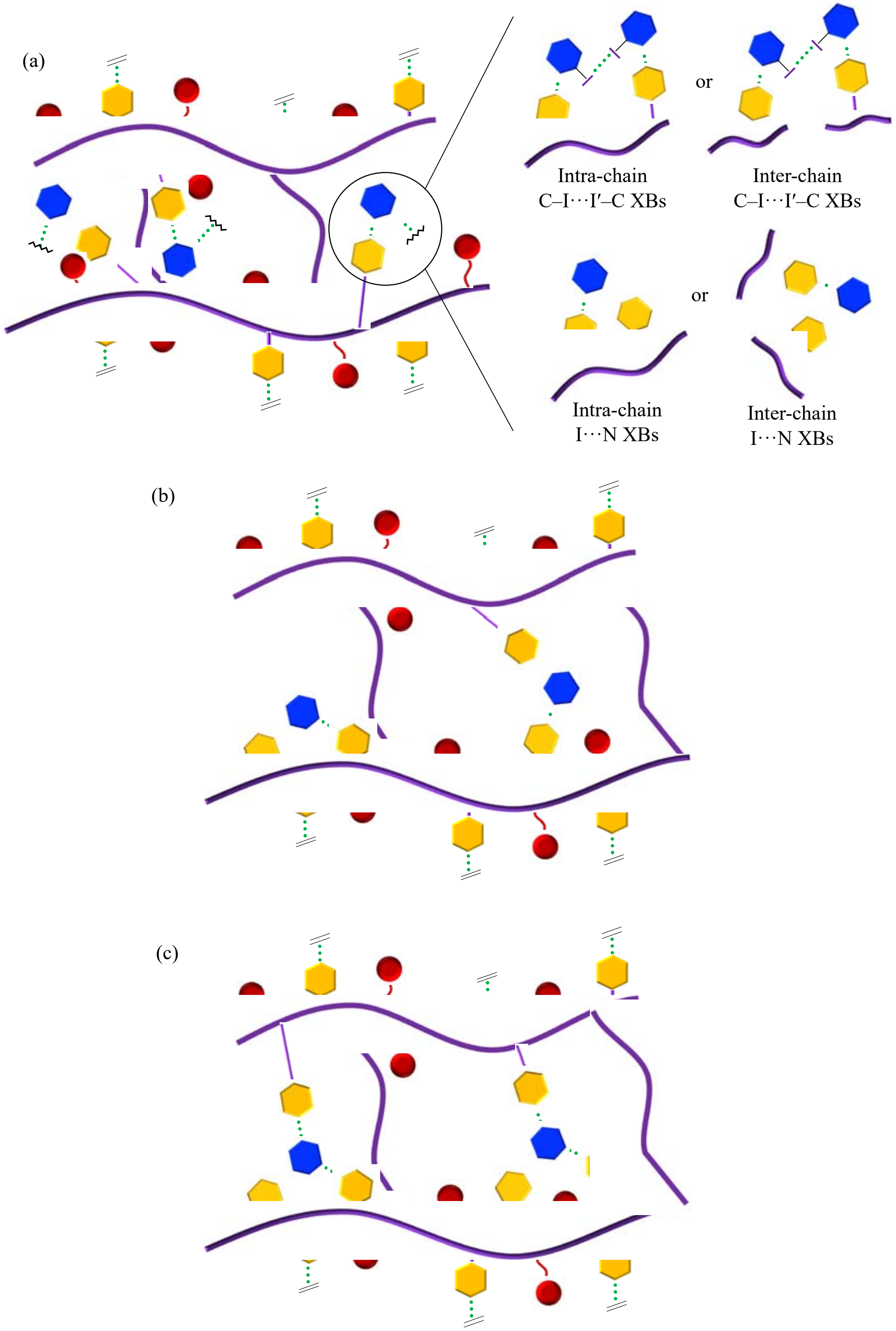

Supplementary Figure 14. Predicted halogen-bonded polymeric structure of (a) **PD$_6$**, (b) **PD$_7$**, (c) **PD$_8$**.

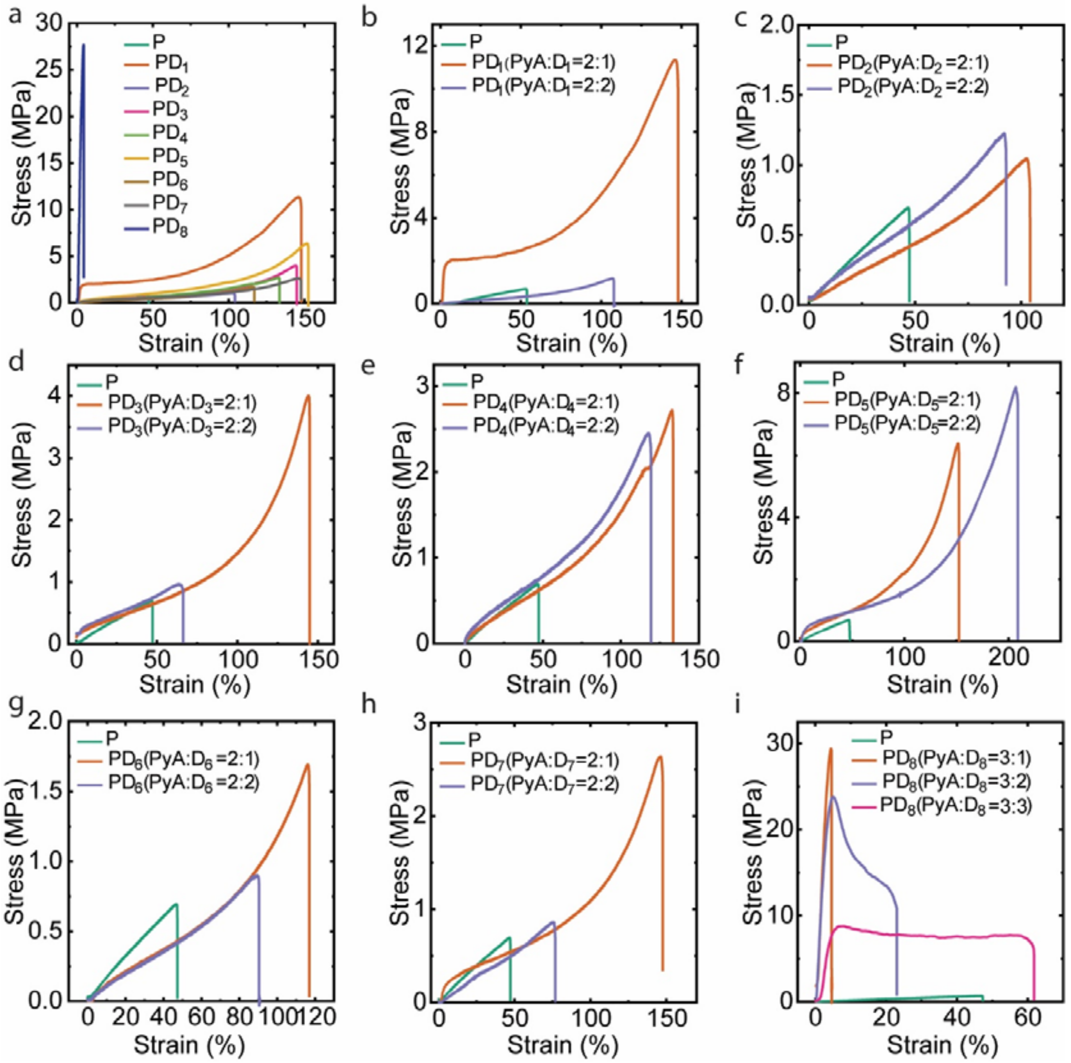

Supplementary Figure 15. (a) Stress-strain curves of **P** and **PD$_1$-PD$_8$**. (b)-(i) Stress-strain curves of **PD$_1$-PD$_8$** at different PyA:**D$_n$** ratios.

Supplementary Table 2: Mechanical properties of **P** and **PD$_n$** at different PyA:D$_n$ ratios

**Code**	**E_Y (MPa) PyA:D$_n$=2:1**	**E_Y (MPa) PyA:D$_n$=2:2**	**σ_{max} (MPa) PyA:D$_n$=2:1**	**σ_{max} (MPa) PyA:D$_n$=2:2**	**ε_{max} (%) PyA:D$_n$=2:1**	**ε_{max} (%) PyA:D$_n$=2:2**
**P**	1.4±0.1		0.5±0.2		32±12.5	
**PD$_1$**	93.6±14.2	0.7±0.1	10.5±1.1	1.2±0.1	142±5.5	109±12
**PD$_2$**	0.8±0.1	1.2±0.1	0.9±0.1	1.2±0.3	91±12.1	85±16
**PD$_3$**	1.3±0.1	1.1±0.1	3.9±0.2	1.3±0.6	149±5	76±21
**PD$_4$**	1.2±0.1	1.5±0.1	2.9±0.1	2.5±0.2	134±5	115±5
**PD$_5$**	2.2±0.5	2.4±0.5	6.7±1	6.3±1.3	154±2	193±10
**PD$_6$**	0.9±0.1	0.8±0.1	1.5±0.3	0.8±0.2	115±5	81±12
**PD$_7$**	1.1±0.2	1.0±0.1	3±0.7	1±0.1	149±12	80±4
**PD$_8$**	863±45*	440±26.5 (599.3±84.5)◆	26±2.6*	10±2 (22.3±3.8)◆	2.4±0.8*	53±8 (21±3)◆

* PyA:D$_n$ = 3:1; ◆PyA:D$_n$ = 3:2

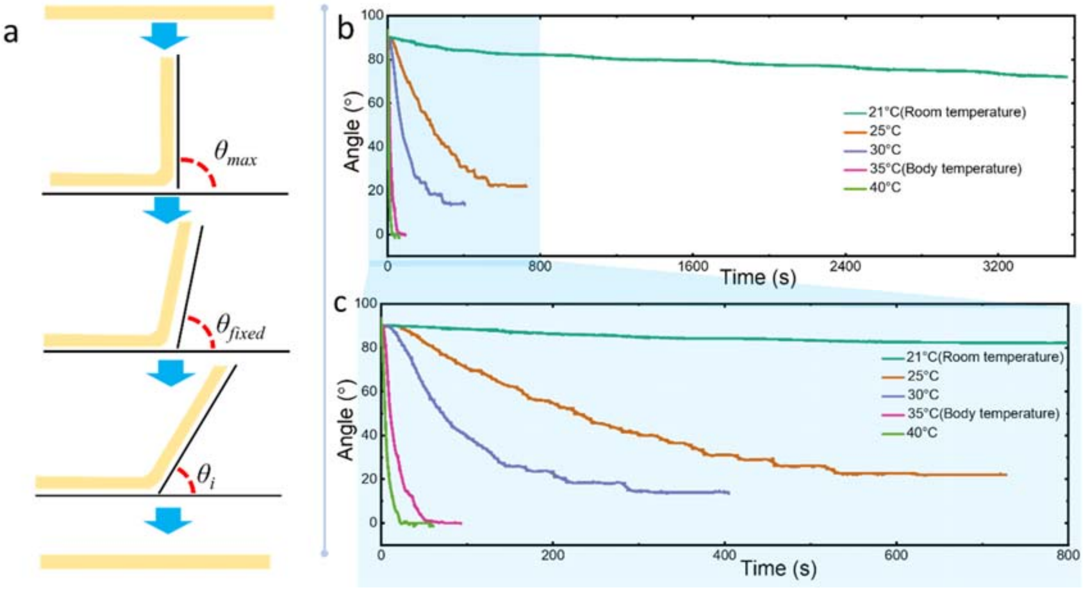

Supplementary Figure 16. Shape memory test. a) Process for testing the shape fixation properties: maximum bending angle and fixed bending angle. b) **PD$_1$** displaying shape recovery curve at different temperatures, and c) zoom-in of (b) shape recovery curve at different temperatures.

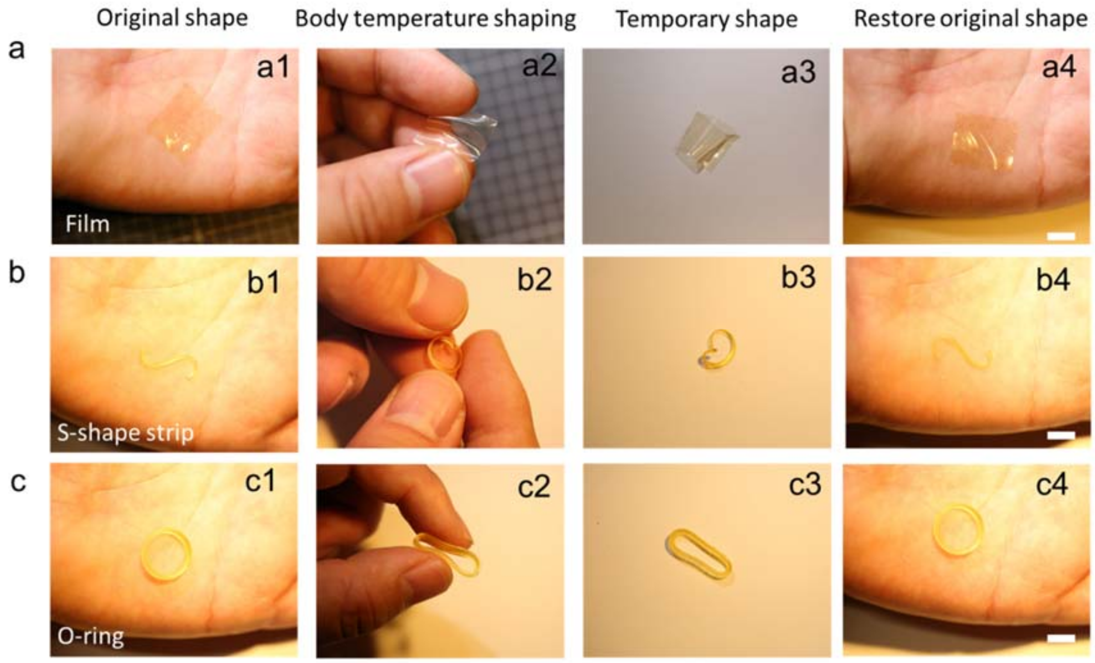

Supplementary Figure 17. Photos showing sample shape programming by hand, and recovery to the original shape on the palm, driven by body temperature. (a) thin film configuration; a1-a4 represent the shape deformation and recovery process of the film. (b) S-shapes strip; b1-b4 represent the shape deformation and recovery process of the strip. (c) O-ring shape; c1-c4 represent the shape deformation and recovery process of the O-ring. Scale bar: 1 cm.

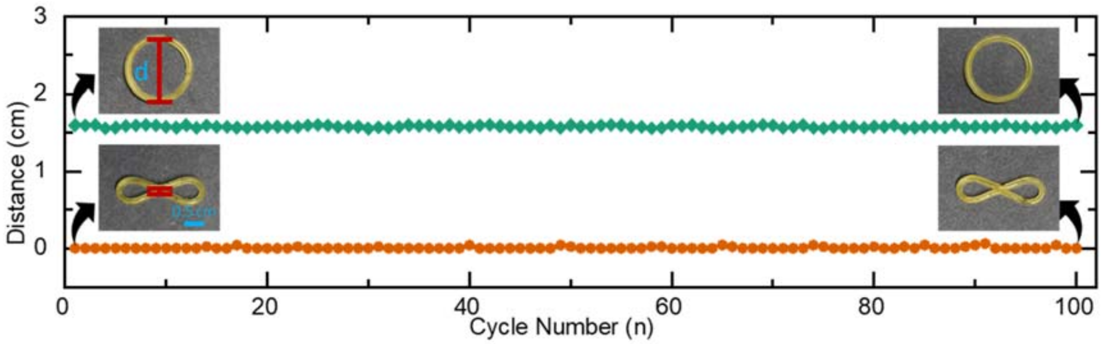

Supplementary Figure 18. Shape memory cycling stability of O-ring made using $\mathbf{PD_1}$

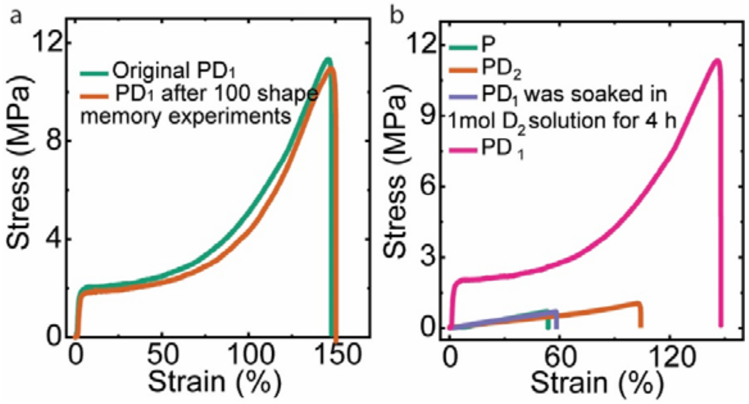

Supplementary Figure 19. (a) Stress-strain curves of **PD_1** before and after 100 shape memory cycles. (b) Stress-strain curves of **P**, **PD_1**, **PD_2** and **PD_1** soaked in D_2 dichloromethane solution for 4 h.

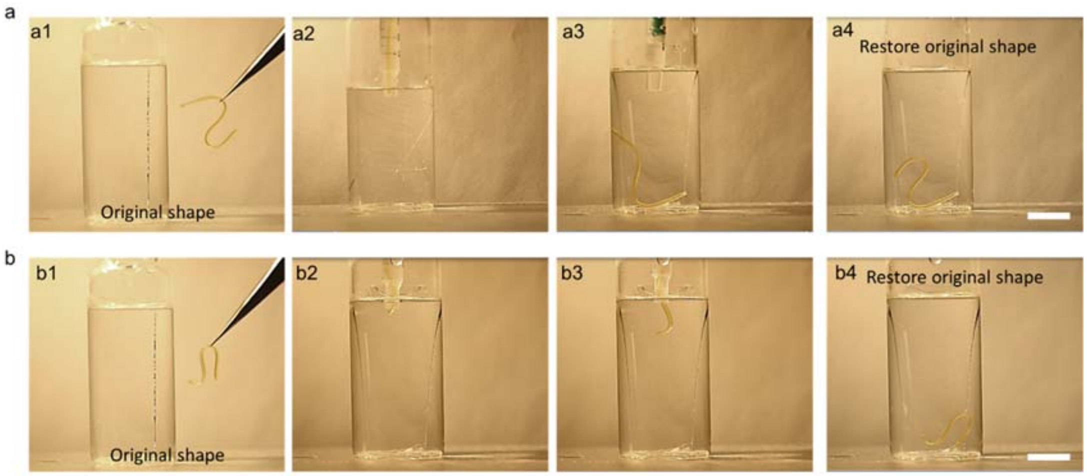

Supplementary Figure 20. Photos showing the shape recovery process upon injection of two samples with different shapes into 37 ºC water to restore their original shape. (a) S-shape; a1-a4 represent the original shape, shape deformation, and shape recovery processes. (b) n-shape; b1-b4 represent the original shape, shape deformation, and shape recovery processes. Scale bar: 1 cm.

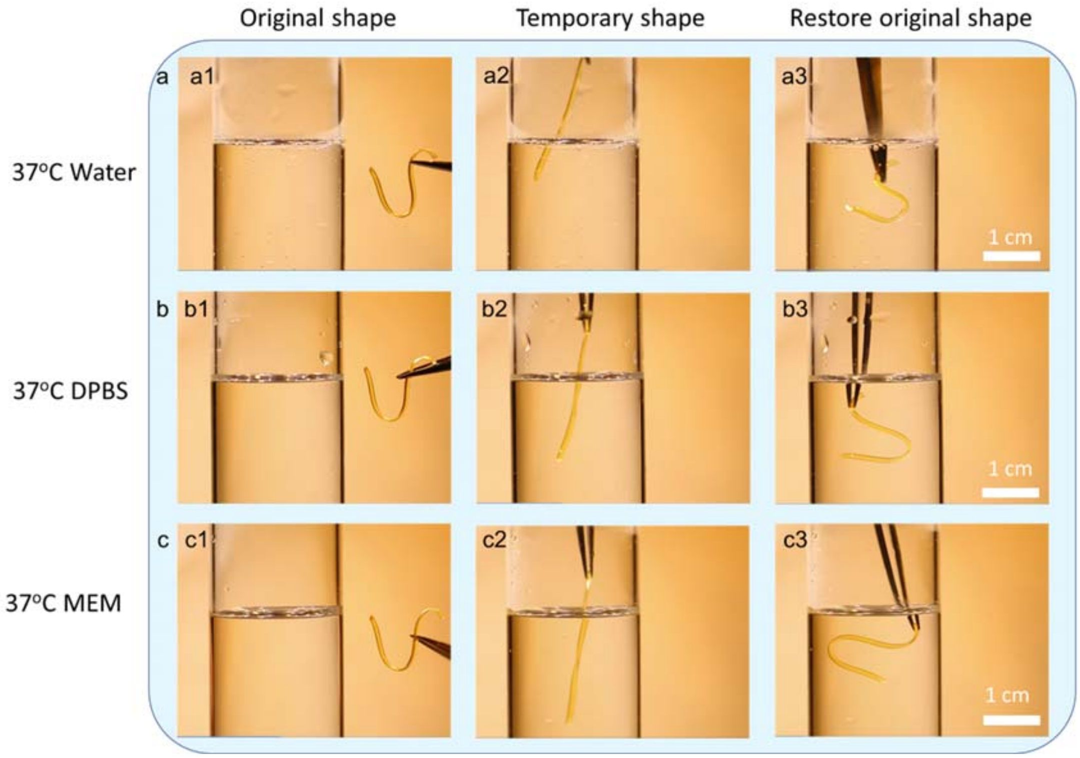

Supplementary Figure 21. Photos showing the shape recovery process in (a) 37ºC water, (b) 37ºC DPBS, and (c) 37ºC MEM. a1, b1 and c1 represent the original shape; a2, b2 and c2 represent the temporary shape; a3, b3 and c3 represent the restored original shape.

Supplementary Note 1 Synthesis of 4-pyridyl methacrylate (**PyA**)

A solution of 4-pyridinemethanol (8.70 g, 79.72 mmol, 1.0 eq) in 350 mL of dichloromethane (DCM) was cooled to 0 °C, and triethylamine (25.0 g, 247.14 mmol, 2.10 eq) was added dropwise over the course of 5 min. Acryloyl chloride (15.15 g, 167.42 mmol, 3.10 eq) in 50 mL of DCM was then added dropwise over the course 60 min, while maintaining the bath temperature below 0 °C. The solution was warmed to room temperature, stirred for 16 hours, and DCM was removed under reduced pressure. The residue is triturated with ethyl acetate (250 mL) and filtered to remove the brown colour solids. The filterate was washed with water (3x100 mL). The organic layer was dried over anhydrous $MgSO_4$, filtered, and removed under reduced pressure to give brown colour liquid. The crude was purified by column chromatography using 8:2 hexane:ethyl acetate as the eluent to yield title compound as fluorescent greenish colour oil (0.867 g, 5.31 mmol, 6.6%). Caution: It is recommended to store PyA at −20 °C. The title compound turns to brown colour liquid at room temperature conditions. ^{1}H NMR (500 MHz, $CDCl_3$): δ = 8.60 (m, 2H), 7.27 (m, 2H), 6.49 (dd, 1H), 6.21 (dd, 1H), 5.93 (dd, 1H), 5.21 (s, 2H) ppm; ^{13}C NMR (125 MHz, $CDCl_3$): δ = 161.1, 149.7, 144.7, 131.8, 121.9, 64.3 ppm.

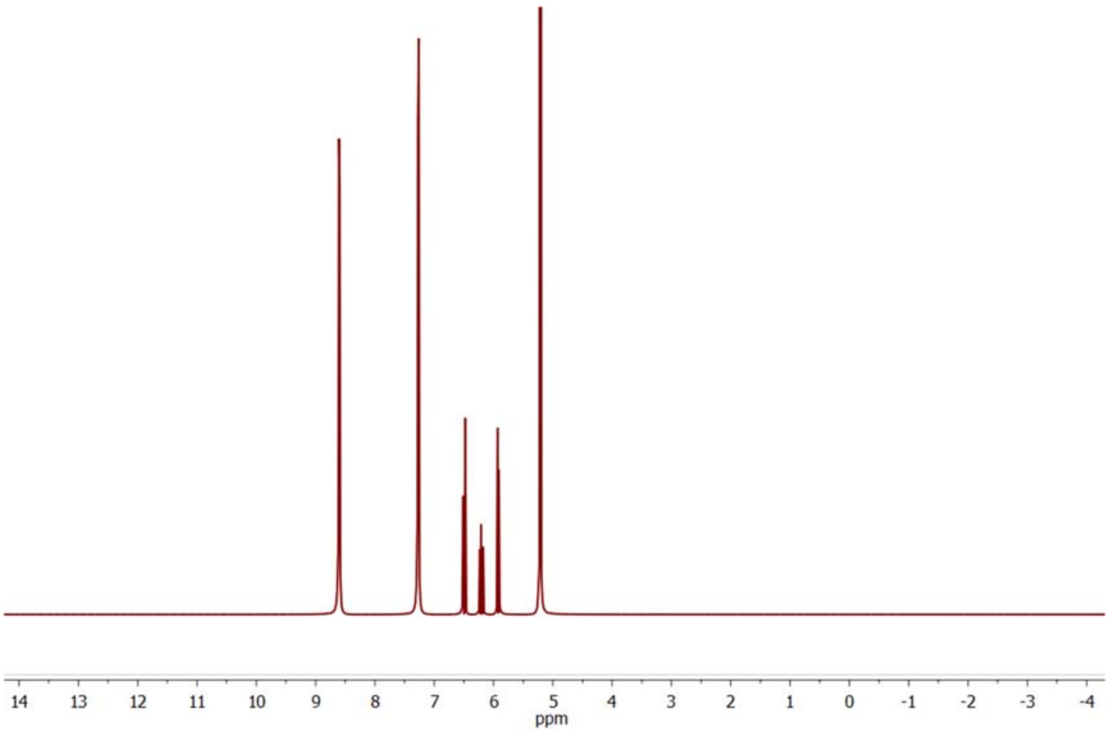

Supplementary Figure 22. ^{1}H NMR (500 MHz, $CDCl_3$) spectrum of PyA.

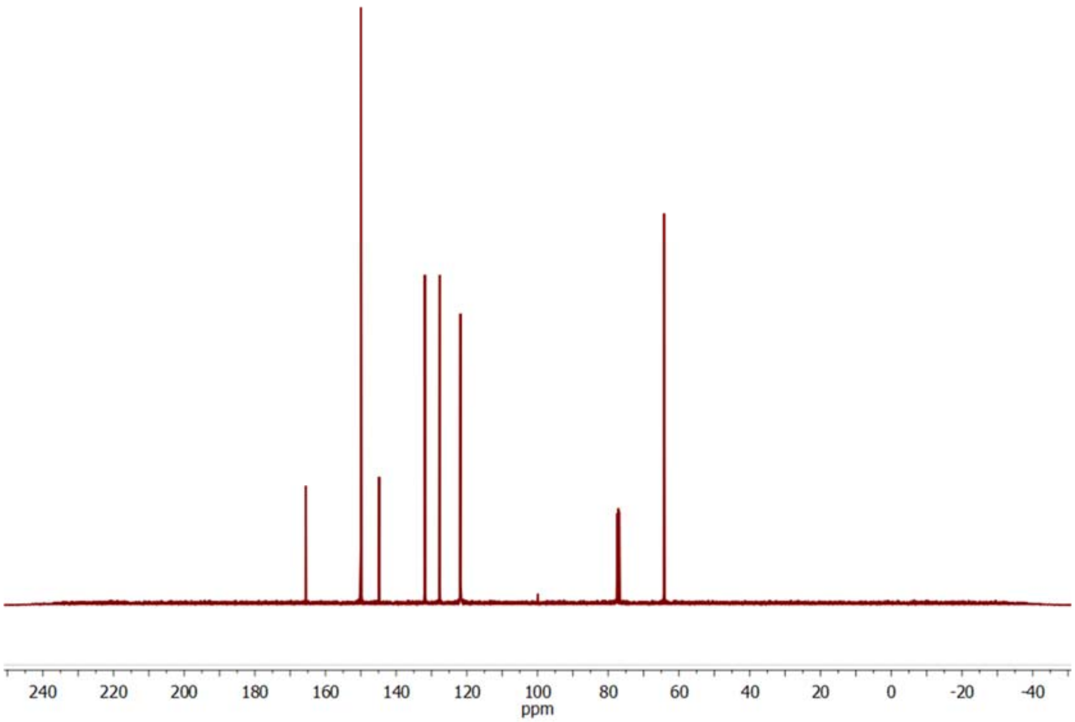

Supplementary Figure 23. ^{13}C NMR (125 MHz, $CDCl_3$) spectrum of PyA.

Supplementary Note 2 Solid state magic-angle spinning (MAS) NMR of the halogen-bonded D_1 complexes.

We carried out solid-state ^{19}F and ^{13}C MAS NMR experiments on an 18.8 T Bruker Avance III HD spectrometer equipped with a 3.2 mm H/F X probe for D_1, **D_1•PyA** and **PD_1** film. The cooling gas temperature was set to –10 °C and the MAS frequency was 16 kHz. While **PD_1** and **D_1•PyA** demonstrated rather broad ^{19}F resonances, probably because of the influence of the structural heterogeneity (low crystallinity), D_1 provides highly resolved NMR signals. We found the chemical shift of the fluorine signal of D_1 in **D_1•PyA**, **PD_1** was shifted to higher field by approximately 5 ppm relative to pure D_1 (see Supplementary Figure 24a).

In ^{19}F-^{13}C cross polarisation (CP)-MAS spectra (see Supplementary Figure 24b), we observed a high-field chemical shift of 1.30 ppm for C–F of D_1 in the **PD_1** complex relative to the uncomplexed D_1. The **D_1•PyA** sample gave very low signal intensity, however, a broad signal at higher fields relative to uncomplexed D_1 was visible. We believe this shift is induced by the I···N XB formation. Similar observations were used to confirm the XB complexation involving 1,4-diiodotetrafluorobenzene and 3-nitropyridine in the literature.[1] We did not observe the C–I signal in the **PD_1** and **D_1•PyA** spectra.

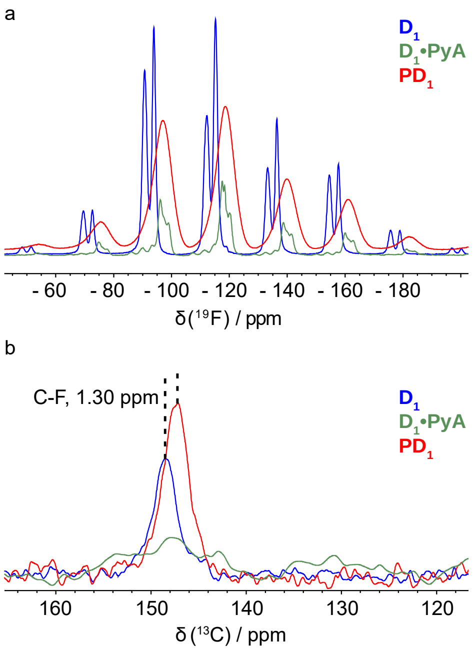

Supplementary Figure 24. Solid-state MAS NMR results. (a) Overlay of ^{19}F NMR spectra of D_1, **D_1•PyA** and **PD_1**. The chemical shifts were referenced externally relative to LiF. (b) Overlay ^{19}F-^{13}C CP-MAS NMR spectra of D_1, **D_1•PyA** and **PD_1** acquired using a Hahn-echo sequence. The chemical shifts were referenced externally relative to adamantane.

1 Hajjar, C., Nag, T., Sayed, H. A., Ovens, J. S. & Bryce, D. L. Stoichiomorphic halogen-bonded cocrystals: a case study of 1,4-diiodotetrafluorobenzene and 3-nitropyridine. *Can. J. Chem.* **100**, 245-251 (2022).
