# Supplementary material for: Halogen-bonded shape memory polymers
Source: Nat Commun. 2022 Dec 5;13:7436. doi: 10.1038/s41467-022-34962-7 (PMC9723116; doi:10.1038/s41467-022-34962-7)
Supplement: Supplementary file 2 — Description of Additional Supplementary Files [file 41467_2022_34962_MOESM2_ESM.pdf]

Description of Additional Supplementary Files

File Name: Supplementary Movie 1
Description: **Shape programming with hand**. A 100 µm PD_1 film is manually shaped into an irregular shape. The surface of the hand is roughly 35 ^oC hot.

File Name: Supplementary Movie 2
Description: **Restoring the film's original shape on a human palm.** A 100 µm PD_1 film returns to its original shape when placed on human hand. The surface of the hand is roughly 35 ^oC hot.

File Name: Supplementary Movie 3
Description: **Manual preparation of an S-shaped column.** A PD_1 column with a diameter of 1 mm is shape-programmed by hand. The surface of the hand is roughly 35 ^oC hot.

File Name: Supplementary Movie 4
Description: **Restoring the original shape of the S-shaped column on human palm.** A 1 mm diameter columnar temporary shape returns to its original shape when placed on human hand. The surface of the hand is roughly 35 ^oC hot.

File Name: Supplementary Movie 5
Description: **Molding a PD1 polymer ring with hand.** A ring with a thickness of 2 mm is manually shaped. The surface of the hand is roughly 35 ^oC hot.

File Name: Supplementary Movie 6
Description: **The process of restoring the original shape of the ring on the hand.** In a human hand, a 2 mm thickness ring with a temporary shape is returned to its original shape. The surface of the hand is roughly 35 ^oC hot.

File Name: Supplementary Movie 7
Description: **The injection process of a soft robot.** A 14 cm long fiber with a 1 mm diameter was hand-shaped into a straight wire and placed in 20^oC water to maintain the temporary shape. It was then placed in a glass tube with a diameter 1.4 mm to maintain its temporary shape before being injected into 37^oC water, retaining the original coil shape.
